# Resting-State Functional Magnetic Resonance Imaging and Functional Connectivity Density Mapping in Patients With Optic Neuritis

**DOI:** 10.3389/fnins.2021.718973

**Published:** 2021-10-14

**Authors:** Ke Song, Yong Wang, Mei-Xia Ren, Jiao Li, Ting Su, Si-Yi Chen, Yi Shao, Ya-Li Lv

**Affiliations:** ^1^Department of Equipment, Xi’an People’s Hospital, Xi’an Fourth Hospital, Xi’an, China; ^2^Department of Ophthalmology, Xi’an People’s Hospital, Xi’an Fourth Hospital, Xi’an, China; ^3^Fujian Provincial Key Laboratory of Ophthalmology and Visual Science, Eye Institute of Xiamen University, Medical College of Xiamen University, Xiamen, China; ^4^Department of Ophthalmology, Massachusetts Eye and Ear and Harvard Medical School, Boston, MA, United States; ^5^Department of Ophthalmology, The First Affiliated Hospital of Nanchang University, Nanchang, China; ^6^Department of Neurology, Xi’an People’s Hospital, Xi’an Fourth Hospital, Xi’an, China

**Keywords:** resting-state functional connectivity, brain activities, optic neuritis, functional connectivity density, functional magnetic resonance imaging

## Abstract

**Background:** Using resting-state functional connectivity (rsFC), we investigated alternations in spontaneous brain activities reflected by functional connectivity density (FCD) in patients with optic neuritis (ON).

**Methods:** We enrolled 28 patients with ON (18 males, 10 females) and 24 healthy controls (HCs; 16 males, 8 females). All subjects underwent functional magnetic resonance imaging (fMRI) in a quiet state to determine the values of rsFC, long-range FCD (longFCD), and short-range FCD (IFCD). Receiver operating characteristic (ROC) curves were generated to distinguish patients from HCs.

**Results:** The ON group exhibited obviously lower longFCD values in the left inferior frontal gyrus triangle, the right precuneus and the right anterior cingulate, and paracingulate gyri/median cingulate and paracingulate gyri. The left median cingulate and paracingulate gyri and supplementary motor area (SMA) were also significantly lower. Obviously reduced IFCD values were observed in the left middle temporal gyrus/angular gyrus/SMA and right cuneus/SMA compared with HCs.

**Conclusion:** Abnormal neural activities were found in specific brain regions in patients with ON. Specifically, they showed significant changes in rsFC, longFCD, and IFCD values. These may be useful to identify the specific mechanism of change in brain function in ON.

## Introduction

Optic neuritis (ON) is a common clinical eye disease that causes inflammation of the optic nerve, sudden loss of vision, and pain during eye movement (Toosy et al., 2014). Diagnosis is typically based on history and clinical presentation. There are many ways to treat ON based on etiological classification and clarifying the pathogenic cause. Non-specific ON is mainly treated with hormone drugs to reduce inflammatory edema of the optic nerve as soon as possible to avoid irreversible neuropathy (Shi et al., 2019). Although inflammation therapy can help restore vision in patients with multiple sclerosis, vision does not return to normal in others, and may be accompanied by abnormal color vision and visual field defects. but in some cases, they cannot fully recover. ON was previously considered as a simple retinal disease, but relevant studies have shown that there are abnormalities in the function of the visual cortex of the brain in PATIENTS with ON (Audoin et al., 2006), which makes it necessary for us to study more potential neural mechanisms of ON.

Functional magnetic resonance imaging (fMRI) has been used in ON research. It can be used to study variation in brain functional connectivity (FC) in resting state and provides more information about cortical activity. Compared with conventional MRI, fMRI offers more information about the activity of the cerebral cortex, allows precise localization, does not cause radiation damage, and facilitates the combination of functional and anatomical imaging. When the ON patient is examined, the functional state of the visual cortex and visual pathway can be non-invasively assessed. Today, fMRI has been employed for neurological studies of eye diseases such as corneal ulcer (Zhu et al., 2019), primary angle-closure glaucoma (Huang et al., 2015), and anisometropic amblyopia (Liang et al., 2017). Related resting state studies have tentatively elucidated the functional changes in the brain associated with these eye diseases.

Resting-state functional connectivity (rsFC) analysis is a valid way to measure the temporal correlation between two successive blood oxygen level-dependent signals and to estimate spontaneous brain activity (Liu et al., 2021). We can calculate the long- and short-distance functional connectivity density (longFCD and IFCD) by mapping the whole-brain functional connectivity density (FCD). These measures help assess functional information in the whole brain. FCD can reflect the characteristics of spontaneous neural activity to reveal functional relationships between different brain regions. Greater FCD values for particular voxels indicate that those voxels are functionally connected to a greater number of other brain voxels and suggest that those voxels play more important roles during information processing. RsFC has been utilized to investigate the abnormal FCD changes in a variety of conditions such as Parkinson’s disease (Hu et al., 2017), depression (Maglanoc et al., 2019), and amblyopia (Liang et al., 2017).

Although many previous studies using fMRI found that patients with ON had reduced functional connectivity within the visual system, showed neuronal morphological changes in the ON, there was far less evidence for changes in the neuromechanism of brain in patients with ON. The purpose of this study is to evaluate rsFC in ON patients in a resting state, including longFCD and IFCD value changes. We tried to use the resting FC method to evaluate functional connection density (FCD) in patients with ON, and to determine the abnormal areas of brain activity. Our results may help clarify the potential neural mechanisms of ON.

## Materials and Methods

### Subjects

We collected data from 28 patients with ON (18 males, 10 females) and 24 healthy controls (HCs; 16 males, 8 females). All the subjects underwent fMRI in resting state.

The ON patient inclusion criteria were: (1) acute eye pain and vision loss, (2) vision abnormalities associated with nerve fiber damage, (3) a relative pupillary block or abnormal visual evoked potential, (4) no brain parenchyma abnormalities on MRI, and (5) no other eye disease. The exclusion criteria for patients were: (1) eye injury or surgery, (2) cardiovascular diseases, (3) mental illness (anxiety, depression, etc.), or (4) other problems that may have hindered the acquisition of rsFC and FCD values.

The inclusion criteria for HCs were: (1) no eye disease with best-corrected visual acuity (BCVA) >1.0, (2) no history of addiction, (3) no mental illness, and (4) no contraindications for MRI.

All the methods used in the study were in accordance with medical ethics and the Helsinki declaration. All subjects agreed to participate, knew about the purpose and potential risks, and signed informed consent forms.

#### MRI Data Acquisition

A 3.0-Tesla MR scanner was used for data acquisition. Subjects were asked to remain still during scanning. The following parameters were used for the T1 and T2 sequences: repetition time (TR) 1,900 ms, echo time (TE) 2.26 ms, thickness 1.0 mm, clearance 0.5 mm, field of view (FOV) 250 mm × 250 mm, matrix 256 × 256, flip angle (FA) 9°, and a total of 176 sagittal sections. Next, 240 functional images were obtained using the gradient echo plane image sequence in the static scanning session: TE 30 ms, section clearance 1 mm, matrix 64 × 64, and FA 90°. We collected 35 oblique slices.

### Data Analysis

The resting images were preprocessed using the brain imaging data processing and analysis box (DPABI2.1) based on MATLAB2010a software (MathWorks Inc., Natick, MA, United States). First, the initial 10 volumes were discarded for signal stabilization. Then, the remaining 200 volumes were realigned to the first volume after correcting for the differences in acquisition times, during which the mean frame-wise displacement (FD) was calculated. We used the Friston 24 head motion parameter higher-order model to reduce effects of head movement (Satterthwaite et al., 2013; Yan et al., 2013). Next, the images were normalized into the Montreal Neurological Institute (MNI) space. Finally, 0.01–0.1 Hz band pass filtering was performed for the time series of every voxel to reduce the influence of other factors such as low-frequency drift, respiration, and linear attenuation.

### Long- and Short-Distance Functional Connectivity Density Mapping

Based on the Pearson correlation values of voxel activity, we connected the functions of voxels as a binary chart of node degrees. The MATLAB BrainWave toolbox (MathWorks Inc.) was used to calculate IFCD and longFCD values for each node to estimate several network connectivity measures. Node and local efficiency estimation is an adaptive estimate of the performance of small-world brain function network (Song et al., 2021). A relevant threshold *r* > 0.25 was used to define given voxels with other whole-brain FC values for the voxel (Hata et al., 2016). The IFCD voxels in the adjacent area (anatomical distance ≤14 mm) that met the threshold that mean *r* > 0.25 and distances >14 mm were defined as longFCD. To normalize the data, IFCD and longFCD maps were translated to *Z* scores. Finally, statistical parameter mapping using SPM8 (MathWorks Inc.) was used to apply a 6 mm × 6 mm × 6 mm full-width Gaussian kernel to smooth IFCD and longFCD maps.

### Ophthalmic Testing

The BCVA of right eye was measured for all subjects using the Snellen acuity chart at a distance of 5 m with the line of sight parallel to line 1.0.

### Statistical Analysis

SPSS 16.0 software (SPSS, Chicago, IL, United States) was used to analyze the clinical variables. The values of rsFC, longFCD, and IFCD in the ON and HC groups were tested with independent-sample *t*-tests. Two-sample *t*-tests were used to evaluate differences between IFCD and longFCD maps (voxel level *P* < 0.01, corrected by GRF). The mean FD calculated during the preprocessing step was accounted for by including this term as a covariate. The linear model was built using the SPM8 toolkit. The receiver operating characteristic (ROC) curve method was applied to analyze between-group differences in brain regions of average rsFC, longFCD, and IFCD. Pearson correlations were used to assess the relationship between ON patient clinical characteristics and the rsFC, longFCD, and IFCD values. For all analyses, differences were considered significant at *P* < 0.05.

## Results

### Demographic and Visual Measurements

There were no significant differences in weight or age between the ON and HC groups. As expected, significant differences were observed in BCVA between groups. The mean time of ON was 46.32 ± 5.65 days in the patient group (Table 1). Due to the uncertainty of patients’ visit time, we did not include factors such as the length of disease in the study scope.

**TABLE 1:** Participant characteristics.

**Condition**	**ON**	**HCs**	***t***	***P*-value**
Male/female	18/10	16/8	0.664	0.754
Age (years)	54.12 ± 5.58	51.36 ± 5.87	0.321	0.917
Weight (kg)	63.46 ± 7.59	61.19 ± 6.54	0.176	0.929
Handedness	28R	24R	0.215	0.912
Duration of ON (days)	46.32 ± 5.65	N/A	N/A	N/A
BCVA-left eye	0.35 ± 0.15	0.95 ± 015	–3.624	0.014
BCVA-right eye	0.25 ± 0.12	1.10 ± 0.15	–3.226	0.009
IOP-L	16.38 ± 6.78	15.52 ± 616	0.716	0.902
IOP-R	15.22 ± 6.51	16.35 ± 6.27	0.659	0.921

*Independent *t*-tests comparing the two groups (*P* < 0.05 is significant). Data are shown as mean ± SD or *n*.*

*BCVA, best-corrected visual acuity; HCs, healthy controls; IOP, intraocular pressure; L, left; N/A, not applicable; ON, optic neuritis; R, right.*

### Functional Connectivity Density Analysis

**FIGURE 1:**
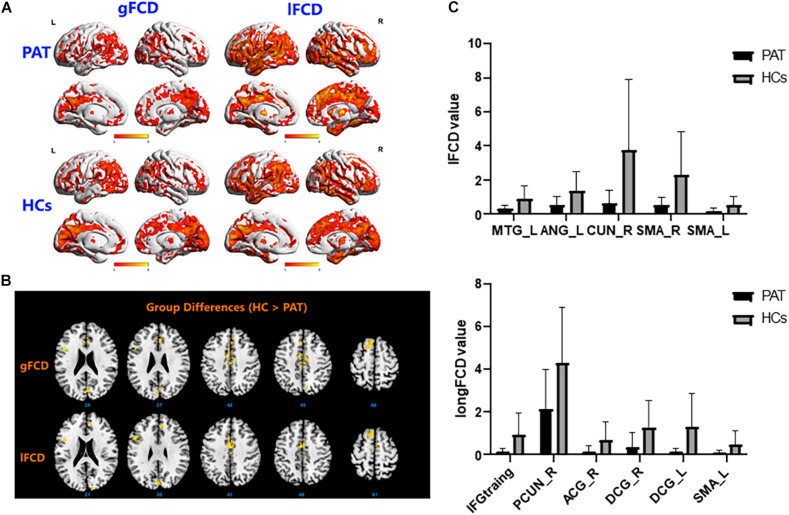
**(A)** Binarized longFCD (left) and IFCD (right) values in different brain regions. **(B)** Binarized longFCD and IFCD differences between the HC and ON groups. Yellow areas show lower values in ON groups. **(C)** Significant IFCD differences were observed in the left middle temporal gyrus/angular gyrus/supplementary motor area/right cuneus/supplementary motor area. Significant longFCD differences were observed in the left inferior frontal gyrus, triangular part/right precuneus/right anterior cingulate, paracingulate gyri/median cingulate, paracingulate gyri/left median cingulate, and paracingulate gyri/supplementary motor area. longFCD, long-range functional connectivity density; IFCD, short-range functional connectivity density; PAT, patient; HCs, healthy controls; L, left; R, right.

The ON group presented with obviously lower longFDC values in the triangular part of the left inferior frontal gyrus (IFGtriang), right precuneus (PCUN), and right anterior cingulate and paracingulate gyri/median cingulate and paracingulate gyri (ACG/DCG). The left median cingulate and paracingulate gyri (DCG) and supplementary motor area (SMA) values were also significantly lower (Table 2 and Figure 1). Moreover, significantly reduced IFCD values were detected in the left middle temporal gyrus (MTG)/angular gyrus (ANG)/SMA and the right cuneus (CUN)/SMA (Table 3 and Figure 1).

**TABLE 2:** The binarized longFCD differences between the ON and HC groups.

**Brain areas**	**MNI coordinates**			**longFCD**		
	***X***	***Y***	***Z***	**BA**	**Peak voxels**	***t*-Value**
HC > PAT						
Frontal_Inf_Tri_L	−45	12	24	44	31	4.14
Precuneus_R	3	−72	45	7	50	3.43
Cingulum_Ant_R	6	30	27	2	32	3.45
Cingulum_Mid_R	6	−27	42	24	51	3.29
Cingulum_Mid_L	−6	−3	42	24	43	4.01
Supp_Motor_Area_L	−6	24	57	6	47	3.34

*Between-group differences in binarized longFCD at a threshold of *r* = 0.3. Voxel-wise *P* < 0.01 and cluster-level *P* < 0.05 were used to identify significant group differences, correcting for multiple comparisons by AlphaSim.*

*BA, Brodmann’s area; longFCD, long-range functional connectivity density; MNI, Montreal Neurological Institute; Pt, patient.*

**TABLE 3:** Binarized IFCD differences between the ON and HC groups.

**Brain areas**	**MNI coordinates**			**lFCD**		
	***X***	***Y***	***Z***	**BA**	**Peak voxels**	***t*-Value**
HC > PAT						
Temporal_Mid_L	9	42	21	32	40	3.81
Angular_L	−48	15	30	44	79	3.67
Cuneus_R	12	−90	21	19	42	3.98
Supp_Motor_Area_R	3	3	48	24	85	3.78
Supp_Motor_Area_L	−12	18	63	6	36	3.20

*Between-group differences in binarized IFCD at a threshold of *r* = 0.3. We used thresholds of two-tailed voxel-wise *P* < 0.01 and cluster-level *P* < 0.05, corrected for multiple comparisons by AlphaSim to determine the significant group differences.*

*BA, Brodmann’s area; HC, healthy control; IFCD, short-range functional connectivity density; MNI, Montreal Neurological Institute; ON, optic neuritis; ROI, region of interest.*

### Receiver Operating Characteristic Curve Analysis

**FIGURE 2:**
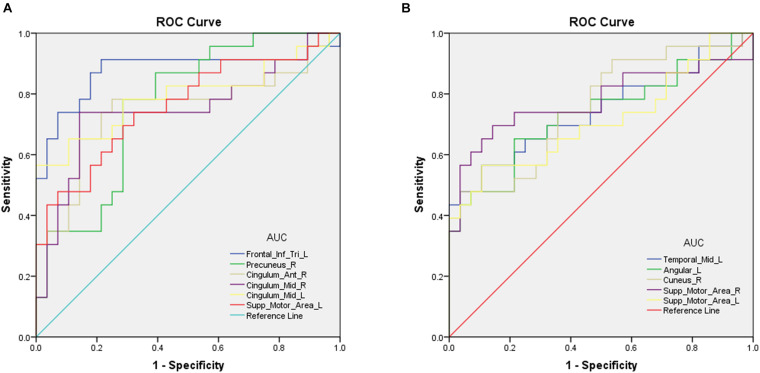
Receiver operating characteristic curve analysis of the mean longFCD **(A)** and IFCD **(B)** values for altered brain regions. **(A)** ROC curve analysis of the gFCD values for altered brain regions. The area under the ROC curve were 0.876 (*P* < 0.001; 95% CI: 0.762–0.990) for Frontal_Inf_Tri_L; Precuneus_R 0.762 (*P* = 0.001; 95% CI: 0.632–0.893), Cingulum_Ant_R 0.747 (*P* = 0.003; 95% CI: 0.599–0.894); Cingulum_Mid_R 0.747 (*P* = 0.003; 95% CI: 0.601–0.893), Cingulum_Mid_L 0.792 (*P* < 0.001; 95% CI: 0.656–0.928), and Supp_Motor_Area_L 0.761 (*P* = 0.001; 95% CI: 0.625–0.896). **(B)** ROC curve analysis of the IFCD values for altered brain regions. The area under the ROC curve were 0.744 (*P* = 0.003; 95% CI: 0.600–0.887) for Temporal_Mid_L, Angular_L 0.733 (*P* = 0.005; 95% CI: 0.588–0.878), Cuneus_R 0.758 (*P* = 0.002; 95% CI: 0.623–0.892), Supp_Motor_Area_R 0.778 (*P* = 0.001; 95% CI: 0.635–0.921), and Supp_Motor_Area_L 0.713 (*P* = 0.010; 95% CI: 0.563–0.862). AUC, area under the curve; IFCD, short-range functional connectivity density; longFCD, long-range functional connectivity density; ROC, receiver operating characteristic.

Differences in rsFC, longFCD, and IFCD values between the ON and HC groups would suggest that they could be used as diagnostic biomarkers. The ROC curve method was used to test this. An area under the curve (AUC) value of 0.5–0.7 indicates low precision, 0.7–0.9 indicates medium precision, and >0.9 indicates high precision. The rsFC analysis results are shown in Figure 2. The AUCs were 0.876 [*P* < 0.001; 95% confidence interval (CI): 0.762–0.990] for the IFGtriang, 0.762 for the right PCUN (*P* = 0.001; 95% CI: 0.632–0.893), 0.747 for the right ACG (*P* = 0.003; 95% CI: 0.599–0.894), 0.747 for the right DCG (*P* = 0.003; 95% CI: 0.601–0.893), 0.792 for the left DCG (*P* < 0.001; 95% CI: 0.656–0.928), and 0.761 for the left SMA (*P* = 0.001; 95% CI: 0.625–0.896) (Figure 2A). The AUCs were 0.744 (*P* = 0.003; 95% CI: 0.600–0.887) for the left MTG, 0.733 for the left ANG (*P* = 0.005; 95% CI: 0.588–0.878), 0.758 for the right CUN (*P* = 0.002; 95% CI: 0.623–0.892), 0.778 for the right SMA (*P* = 0.001; 95% CI: 0.635–0.921), and 0.713 for the left SMA (*P* = 0.010; 95% CI: 0.563–0.862) (Figure 2B).

## Discussion

**FIGURE 3:**
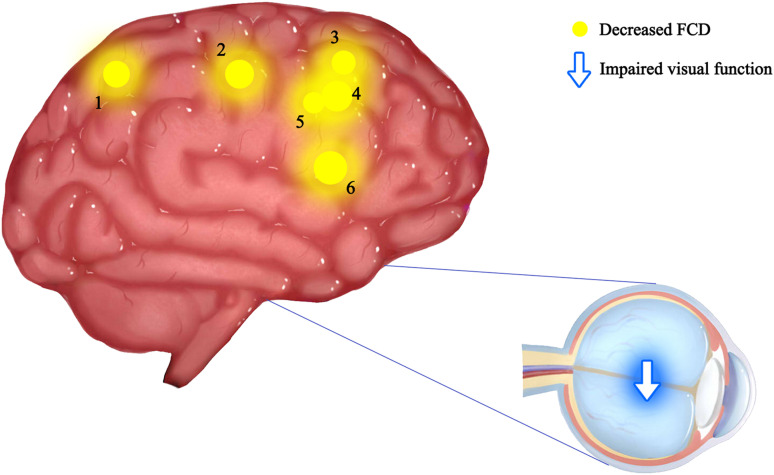
Mean longFCD values of altered brain regions in the ON group. The spot size indicates the extent of change. The longFCD values of the following regions were reduced: 1-inferior frontal gyrus, triangular part (BA44, *t* = –4.14), 2-precuneus (BA7, *t* = –3.43), 3-anterior cingulate and paracingulate gyri (BA2, *t* = –3.45), 4-median cingulate and paracingulate gyri (BA24, *t* = –3.29), 5-median cingulate and paracingulate gyri (BA24, *t* = –4.01), and 6-supplementary motor area (BA6, *t* = –3.34). gFCD, long-range functional connectivity density; PAT, patient; HCs, healthy controls; BA, Brodmann’s area.

**FIGURE 4:**
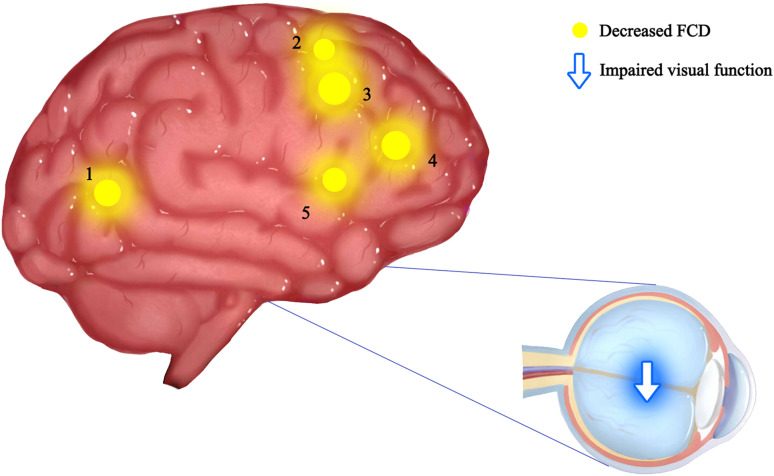
Mean IFCD values of altered brain regions in the ON group. The spot size indicates the extent of change. The IFCD values of the following regions were reduced: 1-middle temporal gyrus (BA32, *t* = –3.81), 2-angular gyrus (BA44, *t* = –3.67), 3-cuneus (BA19, *t* = –3.98), 4-supplementary motor area (BA24, *t* = –3.78), and 5-supplementary motor area (BA6, *t* = –3.20). IFCD, short-range functional connectivity density; PAT, patient; HCs, healthy controls; BA, Brodmann’s area.

The current study investigated the alterations of functional organization for ON patients using a recently developed FCD method. FCD, a reliable rs-fMRI analytical method has been widely used in the study of various ophthalmic and neurological diseases (Wang et al., 2014; Qin et al., 2015; Zhai et al., 2016; Hu et al., 2017; Chen et al., 2019; Li et al., 2019; Dai et al., 2020; Pedersini et al., 2020; Table 4). In addition to impaired visual function, the ON group showed significantly reduced longFCD values compared with HCs in the left IFGtriang, right PCUN, right ACG/DCG, and left DCG/SMA (Figure 3). Obviously decreased IFCD values were also found in the left MTG/ANG/SMA and right CUN/SMA (Figure 4).

**TABLE 4:** Functional connectivity density method applied in ophthalmologic and neurogenic diseases.

	**References**	**Disease**
Ophthalmologic diseases	Zhai et al., 2016	High myopia
	Qin et al., 2015	Congenitally and late blindness
	Wang et al., 2014	Anisometropic amblyopia
	Pedersini et al., 2020	Hemianopia
	Chen et al., 2019	Primary angle-closure glaucoma
Neurogenic diseases	Liu et al., 2021	Heroin users
	Dai et al., 2020	Chronic migraine
	Li et al., 2019	Benign epilepsy with centrotemporal spikes

**FIGURE 5:**
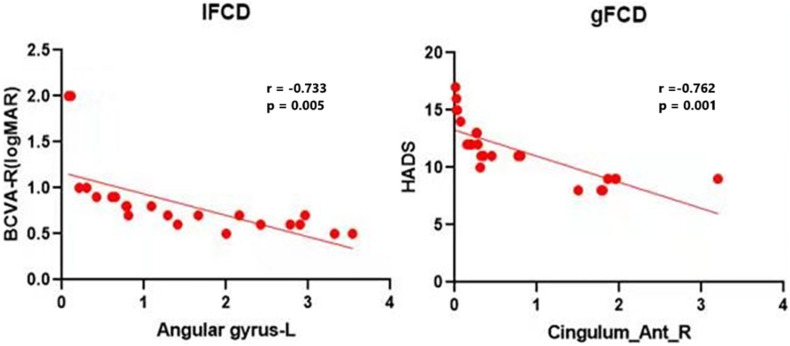
Correlations between the mean IFCD values and BCVA-R (logMAR) and correlations between the mean longFCD values and HADS. There was a negative correlation with the IFCD values of the left angular gyrus and the longFCD values of the right anterior cingulate and paracingulate gyri. IFCD, short-range functional connectivity density; gFCD, long-range functional connectivity density; BCVA-R, best corrected visual acuity of right eye. HADS, Hospital Anxiety and Depression Scale.

A previous study showed that some brain areas show greater activity at rest than during tasks. These areas are important for maintaining brain stability. In the resting state, the brain’s default mode network (DMN) is continuously activated (Song et al., 2021). The DMN encompasses many brain functional areas including the medial frontal lobe, anterior cingulate, inferior temporal lobe, posterior cingulate, PCUN, and inferior parietal lobe (IPL). These brain functional areas have been the focus of default mode research (Raichle et al., 2001). Several studies have shown dysfunction of DMN in Parkinson’s disease (Beason-Held, 2011), Alzheimer’s disease (Yao et al., 2014), schizophrenia (Chang et al., 2015), and depression (Pankow et al., 2015). Toosy et al. (2002) found abnormal activation of the posterior parietal and lateral temporal cortices in ON patients, while Werring et al. (2000) found similar activation in the lateral temporal lobe, posterior parietal cortex, and thalamus. Rocca et al. (2010) reported DMN abnormalities associated with cognitive impairment in ON patients. In support of these findings, we found that the longFCD values of the right PCUN, bilateral median cingulate gyrus and accessory cingulate gyrus, and the right anterior cingulate gyrus were lower in ON patients (*r* = –0.762, *P* = 0.001, Figure 5). This study showed a significant negative correlation even after removing a large bias data. We speculate that the decreased FCD signal value of the left middle frontal gyrus may reflect the damage to DMN in ON. Collectively, these findings indicate that ON has a negative effect on DMN activity.

Interestingly, the bilateral anterior cingulate/medial frontal back longFCD signal was lower than in other areas. Previous studies associated anterior cingulate cortex dysfunction with pain and depression (Onoda and Yamaguchi, 2015; Russo and Sheth, 2015), there may be abnormal in patients with ON with pain and depression. Therefore, impaired vision in patients with ON may be related to bilateral PCUN dysfunction (Colby et al., 1988; Wang et al., 2008). Further confirmation of the association of ON with depression and pain symptoms will be planned in more detail in subsequent studies. The IPL consists of the lbIPS, AG, and SMG (Zhang and Li, 2014), and the ANG is related to the processing of visual spatial information (Egner et al., 2008). We observed decreased IFCD values in the left ANG. Therefore, we concluded that ON can lead to dysfunction of the left ANG (*r* = −0.733, *P* = 0.005). The decrease in IFCD value reflects the severity of nerve injury (Figure 5). Furthermore, from what we have discussed above, here is a summary of the function of the above cerebral regions and the effects of the corresponding dysfunction (Table 5).

**TABLE 5:** Brain regions alternation and its potential impact.

**Brain regions**	**Experimental result**	**Brain function**
Left triangular inferior frontal gyrus	ON < HCs	Emotional control and cognitive function
Right precuneus	ON < HCs	Visual formation, episodic memory, self-related information processing and consciousness
Right anterior cingulate gyrus	ON < HCs	Social behavior, emotional control, motivation, pain perception, and depression
Bilateral medial cingulate gyrus	ON < HCs	Emotional control, self-evaluation, and depression
Bilateral supplementary motor area	ON < HCs	Maintain the simple movement of the body
Left middle temporal gyrus	ON < HCs	Memory function, speech function, somatosensory disorder, depression, and anxiety
Left angular gyrus	ON < HCs	Visual language center and language reading function

*HCs, healthy controls; ON, optic neuritis.*

The present study has several limitations. First, the sample was relatively small. The second reason is that ON is an inflammation that varies significantly in duration and severity. In the following study, we will expand the sample size and adopt more rigorous evaluation methods.

## Conclusion

In summary, we found that ON patients showed abnormal neural activity in specific brain areas, including significant changes in binary rsFC, longFCD, and IFCD values. These findings may help identify specific mechanisms underlying changes in functional brain activity in patients with ON, to provide assistance for the diagnosis of ON. Some limitations exist in our study, for example, the sample size was relatively small although available results have shown some correlation and we did not assess patients before and after treatment. We hope to investigate the effect of treatment of larger samples in future studies.

## Data Availability Statement

The raw data supporting the conclusions of this article will be made available by the authors, without undue reservation.

## Ethics Statement

The studies involving human participants were reviewed and approved by the Xi’an People’s Hospital, Xi’an Fourth Hospital. The patients/participants provided their written informed consent to participate in this study.

## Author Contributions

KS performed all data analysis and wrote the manuscript. YW, TS, and S-YC raised the conception of the study. M-XR and JL contributed the collection of MRI data. YS and Y-LL contributed the manuscript revision. All authors read and approved the submitted version.

## Conflict of Interest

The authors declare that the research was conducted in the absence of any commercial or financial relationships that could be construed as a potential conflict of interest.

## Publisher’s Note

All claims expressed in this article are solely those of the authors and do not necessarily represent those of their affiliated organizations, or those of the publisher, the editors and the reviewers. Any product that may be evaluated in this article, or claim that may be made by its manufacturer, is not guaranteed or endorsed by the publisher.

## References

[B1] Audoin B. Fernando K. T. Swanton J. K. Thompson A. J. Plant G. T. Miller D. H. (2006). Selective magnetization transfer ratio decrease in the visual cortex following optic neuritis. *Brain* 129 1031–1039. 16495327 10.1093/brain/awl039

[B2] Beason-Held L. L. (2011). Dementia and the default mode. *Curr. Alzheimer Res.* 8 361–365. 21222595 10.2174/156720511795745294 PMC3134578

[B3] Chang Y.-T. Huang C.-W. Chang Y.-H. Chen N.-C. Lin K.-J. Yan T.-C. (2015). Amyloid burden in the hippocampus and default mode network: relationships with gray matter volume and cognitive performance in mild stage Alzheimer disease. *Medicine* 94:e763. 10.1097/MD.0000000000000763 25906109 PMC4602683

[B4] Chen L. Li S. Cai F. Wu L. Gong H. Pei C. (2019). Altered functional connectivity density in primary angle-closure glaucoma patients at resting-state. *Quant. Imaging Med. Surg.* 9 603–614. 10.21037/qims.2019.04.13 31143651 PMC6511722

[B5] Colby C. L. Gattass R. Olson C. R. Gross C. G. (1988). Topographical organization of cortical afferents to extrastriate visual area PO in the macaque: a dual tracer study. *J. Comp. Neurol.* 269 392–413. 2453534 10.1002/cne.902690307

[B6] Dai L. Yu Y. Zhao H. Zhang X. Su Y. Wang X. (2020). Altered local and distant functional connectivity density in chronic migraine: a resting-state functional MRI study. *Neuroradiology* 63 555–562. 10.1007/s00234-020-02582-x 33057747

[B7] Egner T. Monti J. M. P. Trittschuh E. H. Wieneke C. A. Hirsch J. Mesulam M. M. (2008). Neural integration of top-down spatial and feature-based information in visual search. *J. Neurosci.* 28 6141–6151. 10.1523/JNEUROSCI.1262-08.2008 18550756 PMC6670545

[B8] Hata M. Kazui H. Tanaka T. Ishii R. Canuet L. Pascual-Marqui R. D. (2016). Functional connectivity assessed by resting state EEG correlates with cognitive decline of Alzheimer’s disease - An eLORETA study. *Clin. Neurophysiol.* 127 1269–1278. 10.1016/j.clinph.2015.10.030 26541308

[B9] Hu X. Jiang Y. Jiang X. Zhang J. Liang M. Li J. (2017). Altered Functional Connectivity Density in Subtypes of Parkinson’s Disease. *Front. Hum. Neurosci.* 11:458. 10.3389/fnhum.2017.00458 28970788 PMC5609108

[B10] Huang X. Cai F.-Q. Hu P.-H. Zhong Y.-L. Zhang Y. Wei R. (2015). Disturbed spontaneous brain-activity pattern in patients with optic neuritis using amplitude of low-frequency fluctuation: a functional magnetic resonance imaging study. *Neuropsychiatr. Dis. Treat.* 11 3075–3083. 10.2147/NDT.S92497 26719692 PMC4689287

[B11] Li R. Wang L. Chen H. Guo X. Liao W. Tang Y.-L. (2019). Abnormal dynamics of functional connectivity density in children with benign epilepsy with centrotemporal spikes. *Brain Imaging Behav.* 13 985–994. 10.1007/s11682-018-9914-0 29956102

[B12] Liang M. Xie B. Yang H. Yin X. Wang H. Yu L. (2017). Altered interhemispheric functional connectivity in patients with anisometropic and strabismic amblyopia: a resting-state fMRI study. *Neuroradiology* 59 517–524. 10.1007/s00234-017-1824-0 28341991

[B13] Liu S. Wang S. Zhang M. Xu Y. Shao Z. Chen L. (2021). Brain responses to drug cues predict craving changes in abstinent heroin users: a preliminary study. *Neuroimage* 237:118169. 10.1016/j.neuroimage.2021.118169 34000396

[B14] Maglanoc L. A. Landrø N. I. Jonassen R. Kaufmann T. Córdova-Palomera A. Hilland E. (2019). Data-Driven Clustering Reveals a Link Between Symptoms and Functional Brain Connectivity in Depression. *Biol. Psychiatry Cogn. Neurosci. Neuroimaging* 4 16–26. 10.1016/j.bpsc.2018.05.005 29980494

[B15] Onoda K. Yamaguchi S. (2015). Dissociative contributions of the anterior cingulate cortex to apathy and depression: topological evidence from resting-state functional MRI. *Neuropsychologia* 77 10–18. 10.1016/j.neuropsychologia.2015.07.030 26235668

[B16] Pankow A. Deserno L. Walter M. Fydrich T. Bermpohl F. Schlagenhauf F. (2015). Reduced default mode network connectivity in schizophrenia patients. *Schizophr. Res.* 165 90–93. 10.1016/j.schres.2015.03.027 25892719

[B17] Pedersini C. A. Guàrdia-Olmos J. Montalà-Flaquer M. Cardobi N. Sanchez-Lopez J. Parisi G. (2020). Functional interactions in patients with hemianopia: a graph theory-based connectivity study of resting fMRI signal. *PLoS One* 15:e0226816. 10.1371/journal.pone.0226816 31905211 PMC6944357

[B18] Qin W. Xuan Y. Liu Y. Jiang T. Yu C. (2015). Functional Connectivity Density in Congenitally and Late Blind Subjects. *Cereb. Cortex* 25 2507–2516. 10.1093/cercor/bhu051 24642421

[B19] Raichle M. E. MacLeod A. M. Snyder A. Z. Powers W. J. Gusnard D. A. Shulman G. L. (2001). A default mode of brain function. *Proc. Natl. Acad. Sci. U. S. A.* 98 676–682. 11209064 10.1073/pnas.98.2.676 PMC14647

[B20] Rocca M. A. Valsasina P. Absinta M. Riccitelli G. Rodegher M. E. Misci P. (2010). Default-mode network dysfunction and cognitive impairment in progressive MS. *Neurology* 74 1252–1259. 10.1212/WNL.0b013e3181d9ed91 20404306

[B21] Russo J. F. Sheth S. A. (2015). Deep brain stimulation of the dorsal anterior cingulate cortex for the treatment of chronic neuropathic pain. *Neurosurg. Focus* 38:E11. 10.3171/2015.3.FOCUS1543 26030699

[B22] Satterthwaite T. D. Elliott M. A. Gerraty R. T. Ruparel K. Loughead J. Calkins M. E. (2013). An improved framework for confound regression and filtering for control of motion artifact in the preprocessing of resting-state functional connectivity data. *Neuroimage* 64 240–256. 10.1016/j.neuroimage.2012.08.052 22926292 PMC3811142

[B23] Shi W.-Q. Liu J.-X. Yuan Q. Ye L. Su T. Jiang N. (2019). Alternations of interhemispheric functional connectivity in corneal ulcer patients using voxel-mirrored homotopic connectivity: a resting state fMRI study. *Acta Radiol.* 60 1159–1166. 10.1177/0284185118815308 30482026

[B24] Song K. Li J. Zhu Y. Ren F. Cao L. Huang Z. G. (2021). Altered Small-World Functional Network Topology in Patients with Optic Neuritis: a Resting-State fMRI Study. *Dis. Markers.* 2021:9948751. 10.1155/2021/9948751 34221189 PMC8219459

[B25] Toosy A. T. Mason D. F. Miller D. H. (2014). Optic neuritis. *Lancet Neurol.* 13 83–99. 10.1016/S1474-4422(13)70259-X 24331795

[B26] Toosy A. T. Werring D. J. Bullmore E. T. Plant G. T. Barker G. J. Miller D. H. (2002). Functional magnetic resonance imaging of the cortical response to photic stimulation in humans following optic neuritis recovery. *Neurosci. Lett.* 330 255–259. 12270641 10.1016/s0304-3940(02)00700-0

[B27] Wang K. Jiang T. Yu C. Tian L. Li J. Liu Y. (2008). Spontaneous activity associated with primary visual cortex: a resting-state FMRI study. *Cereb. Cortex* 18 697–704. 17602140 10.1093/cercor/bhm105

[B28] Wang T. Li Q. Guo M. Peng Y. Li Q. Qin W. (2014). Abnormal functional connectivity density in children with anisometropic amblyopia at resting-state. *Brain Res.* 1563 41–51. 10.1016/j.brainres.2014.03.015 24661911

[B29] Werring D. J. Bullmore E. T. Toosy A. T. Miller D. H. Barker G. J. MacManus D. G. (2000). ZRecovery from optic neuritis is associated with a change in the distribution of cerebral response to visual stimulation: a functional magnetic resonance imaging study. *J. Neurol. Neurosurg. Psychiatry* 68 441–449. 10727479 10.1136/jnnp.68.4.441 PMC1736877

[B30] Yan C.-G. Cheung B. Kelly C. Colcombe S. Craddock R. C. Di Martino A. (2013). A comprehensive assessment of regional variation in the impact of head micromovements on functional connectomics. *Neuroimage* 76 183–201. 10.1016/j.neuroimage.2013.03.004 23499792 PMC3896129

[B31] Yao N. Shek-Kwan Chang R. Cheung C. Pang S. Lau K. K. Suckling J. (2014). The default mode network is disrupted in Parkinson’s disease with visual hallucinations. *Hum. Brain Mapp.* 35 5658–5666. 10.1002/hbm.22577 24985056 PMC4657500

[B32] Zhai L. Li Q. Wang T. Dong H. Peng Y. Guo M. (2016). Altered functional connectivity density in high myopia. *Behav. Brain Res.* 303 85–92. 10.1016/j.bbr.2016.01.046 26808608

[B33] Zhang S. Li C.-S. R. (2014). Functional clustering of the human inferior parietal lobule by whole-brain connectivity mapping of resting-state functional magnetic resonance imaging signals. *Brain Connect.* 4 53–69. 10.1089/brain.2013.0191 24308753 PMC3929139

[B34] Zhu F. Tang L. Zhu P. Lin Q. Yuan Q. Shi W. (2019). Resting-state functional magnetic resonance imaging (fMRI) and functional connectivity density mapping in patients with corneal ulcer. *Neuropsychiatr. Dis. Treat.* 15 1833–1844. 10.2147/NDT.S210658 31308676 PMC6617566

